# Tumoral calcinosis in the cervical spine: a case report and review of the literature

**DOI:** 10.1186/s13256-017-1474-1

**Published:** 2017-10-27

**Authors:** Rui Guo, Tatsuya Kurata, Tetsushi Kondo, Takao Imanishi, Tetsutaro Mizuno, Toshihiko Sakakibara, Yuichi Kasai

**Affiliations:** 1 Department of Spinal Surgery and Medical Engineering, Mie University Graduate School of Medicine, 2-174 Edobashi, Tsu City, Mie 514-8507 Japan; 2 Department of Orthopaedics, The Third People’s Hospital of Kunshan, Kunshan, Jiangsu China; 3 Department of Orthopaedic Surgery, Sakakibara Onsen Hospital, Tsu City, Mie Japan; 4 Department of Orthopaedic Surgery, Murase Hospital, Suzuka City, Mie Japan

**Keywords:** Tumoral calcinosis, Spine, Renal failure, Dialysis

## Abstract

**Background:**

Tumoral calcinosis is rarely located in spine. A 55-year-old Japanese woman with cervical tumoral calcinosis is presented, along with a review of the literature relating to tumoral calcinosis in the spine. We discussed the etiology, diagnosis, and management of this condition.

**Case presentation:**

We report a case of a patient with cervical tumoral calcinosis with end-stage renal disease. A computed tomography scan showed a lobulated, calcified mass around the right facet joint at the fourth-fifth cervical spine and calcifications were also observed in the right intervertebral foramens at fourth-fifth cervical spine and fifth-sixth cervical spine levels and the anterior wall of the spinal canal. By performing a cervical decompression and stabilization, the patient recovered from her neurological symptoms.

**Conclusions:**

Although tumoral calcinosis is rarely located in the spine, it should be considered in the differential diagnosis of spinal lesions. If a calcified mass causes acute neurological symptoms, resection of the mass is still the most important treatment.

## Background

Tumoral calcinosis (TC) was first presented by Inclan *et al.* in 1943 as a disease with large juxta-articular lobular calcified masses [1]. TC is a pathologic entity characterized by the presence of large calcified masses in periarticular soft tissue [2]. It is usually located at large joints. The most common locations of TC in descending order are the hip, elbow, shoulder, foot, and wrist [3].

TC involving the spine is rare and only 31 cases of spinal TC have been reported in the English literature [4–22]. In this case report, a very rare patient with TC located in the cervical spine was presented and previous case reports with TC in spine summarized.

## Case presentation

A 55-year-old Japanese woman presented with a 3-week history of gradually progressing symptoms of numbness, dull pain, and limited range of motion (ROM) in the right upper extremity. Her medical history included chronic renal failure, treated with peritoneal dialysis for the past approximately 5 years.

A physical examination at the initial visit revealed limited ROM of her right shoulder and neck due to pain. The muscle strength of her right upper extremity on the manual muscle testing was one out of five for the deltoid, one out of five for the biceps brachii, and approximately four out of five for other muscle.

**Fig. 1:**
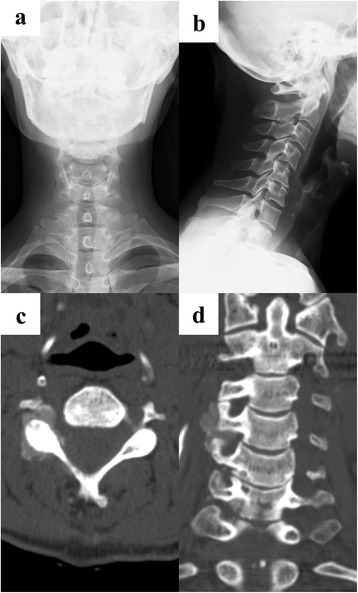
X-ray images show no remarkable change in anteroposterior view (**a**) and slight kyphosis in lateral view (**b**). Axial computed tomography scan image demonstrates lobulated and extraosseous calcified mass in the intervertebral foramen of fourth-fifth cervical spine (**c**). Coronal computed tomography scan image also demonstrates lobulated and extraosseous calcified mass at right side of spine (**d**)

Cervical X-rays (anteroposterior view and lateral view) showed no apparent abnormalities (Fig. 1a, b). Magnetic resonance imaging (MRI) showed slight compression of the dura mater from the right anterior direction of the spinal canal at the fourth-fifth cervical spine (C4-C5) intervertebral level. Her cervical spinal cord was not compressed. Computed tomography (CT) showed a vague calcified lesion around the right facet joint at the C4-C5 level; calcifications were also observed in the right intervertebral foramens at the C4-C5 and C5-C6 levels and the anterior wall of the spinal canal. Calcifications were extraosseous, lobulated, and well-demarcated. There were no destructive changes of cervical vertebrae (Fig. 1c, d). Hematological tests showed renal anemia, increased blood urea nitrogen, creatinine and parathyroid hormone, as well as, slightly increased calcium and phosphorus.

**Fig. 2:**
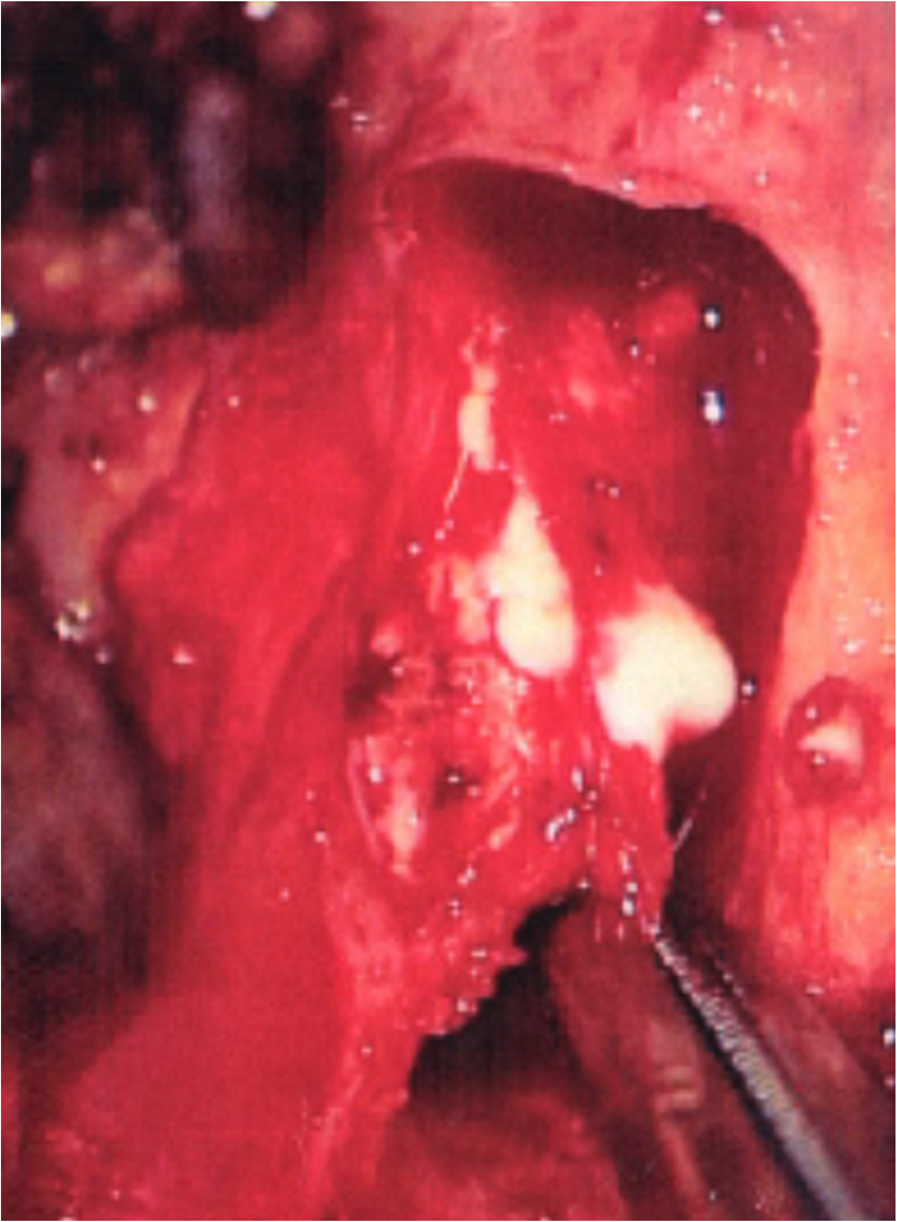
Milky white fluid came out intraoperatively

These findings suggested C5 or C6 radiculopathy due to TC. Surgery was thus performed, with right hemilaminectomy at C4-C5 and C3-6 fusion and internal fixation. When the right C4-C5 facet joint was partly resected, milky white fluid came out (Fig. 2). Around the right-sided nerve root at C5, there was a cyst containing white powder and milky white fluid, with adhesion to the C5 nerve root. Under microscopic observation, this cyst was detached from the nerve root and resected.

**Fig. 3:**
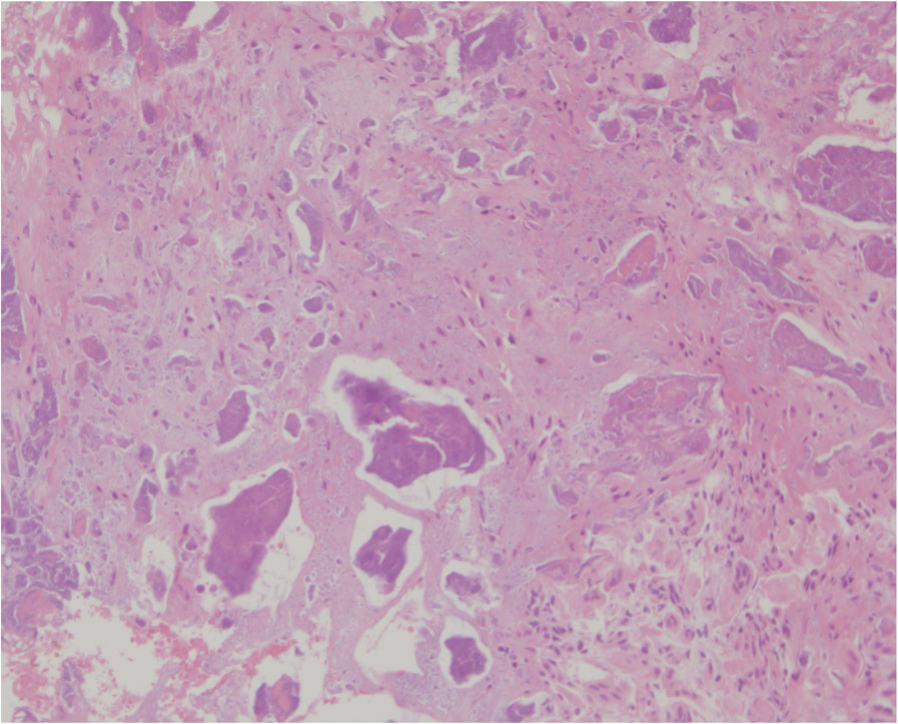
Amorphous calcification and supporting tissue including fibroblasts with few cell components

Histopathological examination of the resected tissue around the white powder showed unstructured (somewhat sand-like granular) materials, which were bluish purple on hematoxylin and eosin staining, along with calcification and supporting tissue including fibroblasts, with few cell components (Fig. 3). The crystals were not composed of calcium pyrophosphate or uric acid.

**Fig. 4:**
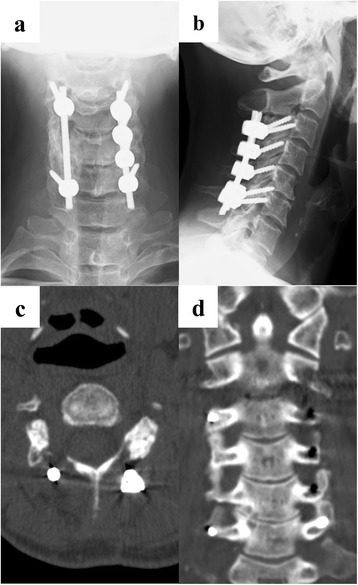
Two-year follow-up images. X-ray images show third-sixth cervical spine C3-C6 fusion and internal fixation: anteroposterior view (**a**), lateral view (**b**). Axial computed tomography scan image demonstrates disappearance of calcified mass in the intervertebral foramen of fourth-fifth cervical spine (**c**). Coronal computed tomography scan image also demonstrates disappearance of calcified mass at right side of spine (**d**)

Her numbness and dull pain in the right upper extremity gradually disappeared postoperatively. The muscle strength of the right upper extremity on the manual muscle testing was full recovered. Postoperative X-rays (anteroposterior view and lateral view) and CT performed 2 years after surgery demonstrated the disappearance of the residual calcified masses (Fig. 4a, b, c, d).

## Discussion

Most cases of TC are located in the shoulder, hip, and metatarsophalangeal joints; they are rarely located in the spine [23]. The common symptoms are local pain, limited ROM of the joint, headache, and numbness and weakness of the extremities. A thorough search of the PubMed database was completed on June 29, 2016. Twenty-eight potentially relevant references, including 48 cases, were identified on the basis of the keywords “tumoral calcinosis” and “spine”. The diagnosis of TC was based on characteristic radiographic features: a calcified, multilobulated, and peripherally corticated mass in the spine. Seventeen cases were excluded, because seven cases with a medical history of scleroderma or rheumatoid arthritis belonged to dystrophic TC, three cases had a final diagnosis of idiopathic TC, and seven cases had no detailed information on treatment. Finally, 32 cases including the present patient were collected. In regard to these patients’ background characteristics, there were 13 males and 19 females, and their mean age was 55.4 years (range 1.4 to 90 years). Overall, 15 cases involved the cervical spine, three involved the thoracic spine, and 14 involved the lumbar spine. The summary of 32 cases is shown in Table 1.

**Table 1:** Summary of reported cases of tumoral calcinosis in spine

Authors and year	Sex	Age (yrs)	Location	Cause	Treatment
Riemenschneider [15] 1952	F	59	L5	NP	Resection, hemilainectomy
Kokubun [11] 1996	F	68	C1-C2	NP	Resection, laminectomy
Mooney [13] 1997	M	1.4	C1-C2	NP	Resection, limited laminectomy
Watanabe [21] 2000	M	55	L4-L5	NP	Resection, laminectomy
Blay [4] 2001	F	44	L5	HP	Medicine therapy
Durant [2] 2001	F	55	L5-S1	Trauma	Resection, hemilainectomy
Durant [2] 2001	M	78	C1-C2	OA	Resection, fusion
Durant [2] 2001	M	64	L4	Surgeries	Resection, arthrodesis
Durant [2] 2001	F	53	T3-T4	NP	Resection, laminectomy
Durant [2] 2001	M	70	L4-L5	OA	Resection, laminoforaminectomy, fusion
Durant [2] 2001	M	55	T4-T5	NP	Laminectomy, vertebrectomy, fusion
Durant [2] 2001	F	54	L3-L4	OA, Surgery	Laminoforaminectomy, fusion
Durant [2] 2001	M	34	L2	Trauma	Fusion
Durant [2] 2001	F	70	C4-C5	NP	Laminectomy
Durant [2] 2001	M	53	L4-L5	NP	Laminectomy
Durant [2] 2001	M	59	L4-L5	Seizures	Laminectomy, facetectomy, foraminotomy
Durant [2] 2001	F	71	L4-L5	NP	Resection, hemilaminectomy, foraminotomy
Iglesias [9] 2002	M	59	T11-T12	NP	Resection, laminectomy
Iglesias [9] 2002	M	55	L5-S1	NP	Resection, laminectomy
Sharma [18] 2005	M	55	L3	NP	Resection, laminectomy
Carlson [5] 2007	F	39	C4-C5	HD	Resection, decompression, arthrodesis, medical control
Wong [22] 2013	F	77	C4-C5	NP	Posterior decompression, arthrodesis
Jackson [10] 2007	F	29	C6-T2	HD	Resection, arthrodesis, total PTX, renal transplantation
Tuy [20] 2008	F	50	C2-C3	HD	Resection
Remy-Leroux [27] 2009	F	29	C6-T1	HD	PTX, renal transplantation
Emon [7] 2011	F	70	L5-S1	SSA	Resection, hemilaminotomy
Matsukado [12] 2001	F	54	C2-C4	HD	Resection, arthrodesis, medical control
Chang [6] 2013	F	44	C1-C2	PD	Resection, laminectomy, arthrodesis
Sunder [28] 2013	F	50	C7-T2	HD	Subtotal PTX, medicine therapy
Sasaki [16] 2015	F	90	C3-C5	NP	Resection, laminectomy
Fatehi [8] 2016	F	73	C2-C3	HD,PD	HD
Our case	M	55	C4-C6	PD	Resection, hemilaminectomy

*M* male, *F* female, *NP* not particular, *HP* hyperphosphatemia, *PD* peritoneal dialysis, *HD* hemodialysis, *SSA* serology negative spondyloarthropathy, *PTX* parathyroidectomy, *OA* osteoarthritis

Smack *et al*. [24] reviewed the literature on TC and proposed a pathogenesis-based classification: primary normophosphatemic TC, primary hyperphosphatemic TC and secondary TC. In our research, 14 cases had no particular causes. One case was related to hyperphosphatemia; 17 cases were secondary TC; four cases had a history of trauma or surgeries to the involved area; nine cases including the present one were related to dialysis; three cases were related to osteoarthritis; and one was related to seronegative spondyloarthropathy.

One of common causes of secondary TC is renal failure. While the etiology of uremic tumoral calcinosis (UTC) is poorly understood, a necessary condition is an elevated serum calcium-phosphate product [25, 26].

Currently, surgical interventions including resection of focal lesions, parathyroidectomy (PTX), and renal transplantation have been performed. Resection is the most important treatment for TC, and it is generally undertaken for lesions causing acute, progressive, or refractory neurological dysfunction. Overall, 21 of 32 cases underwent surgical resections. Chang* et al.* [6] reported a case of UTC causing atlantoaxial subluxation, and they noted that resection and fusion surgery resulted in satisfactory pain relief and functional improvement.

PTX usually achieves remarkable resolution in dialysis patients with severe hyperparathyroidism and elevated serum alkaline phosphatase (ALP) [25]. Chu *et al*. [25] reported three patients who were administered PTX because of co-existing UTC and secondary hyperparathyroidism, but only one case was successfully cured by PTX. The other two patients, who did not have a marked increase in serum ALP preoperatively, had unsatisfactory improvement. Renal transplantation provides complete resolution of UTC [23, 25, 27]. Of the 32 cases, two underwent renal transplantation.

Medical therapy is also used for TC patients, especially for UTC patients using aggressive phosphate binders, calcimimetics, using low-calcium dialysate solutions, and increased length and frequency of hemodialysis treatments [28]. Fatehi *et al.* [8] reported a case that keeping on hemodialysis improved the symptoms and reduced the mass of the lesion.

In this case, a hemilaminectomy of the cervical spine and resection of the calcified masses were performed. CT performed 2 years after surgery demonstrated the disappearance of the residual calcified masses. We report a rare patient with TC located in the cervical spine who was fully recovered neurologically after right hemilaminectomy at C4-C5 and C3-C6 fusion.

## Conclusions

Although TC is rarely located in the spine, it should be considered in the differential diagnosis of spinal lesions. If a calcified mass causes acute neurological symptoms, resection of the mass is still the most important treatment.
